# Glycolysis Changes the Microenvironment and Therapeutic Response Under the Driver of Gene Mutation in Esophageal Adenocarcinoma

**DOI:** 10.3389/fgene.2021.743133

**Published:** 2021-12-08

**Authors:** Lei Zhu, Fugui Yang, Xinrui Li, Qinchuan Li, Chunlong Zhong

**Affiliations:** ^1^ Department of Neurosurgery, Shanghai East Hospital, School of Medicine, Tongji University, Shanghai, China; ^2^ Department of Thoracic Surgery, Shanghai East Hospital, School of Medicine, Tongji University, Shanghai, China; ^3^ Department of Neurology, Shanghai East Hospital, School of Medicine, Tongji University, Shanghai, China

**Keywords:** glycolysis, tumor microenvironment, immunotherapy, drug response, esophageal adenocarcinoma

## Abstract

**Background:** Esophageal cancer is one of the most leading and lethal malignancies. Glycolysis and the tumor microenvironment (TME) are responsible for cancer progressions. We aimed to study the relationships between glycolysis, TME, and therapeutic response in esophageal adenocarcinoma (EAC).

**Materials and Methods:** We used the ESTIMATE algorithm to divide EAC patients into ESTIMATE ^high^ and ESTIMATE ^low^ groups based on the gene expression data downloaded from TCGA. Weighted gene co-expression network analysis (WGCNA) and Gene Set Enrichment Analysis (GSEA) were performed to identify different glycolytic genes in the TME between the two groups. The prognostic gene signature for overall survival (OS) was established through Cox regression analysis. Impacts of glycolytic genes on immune cells were assessed and validated. Next, we conducted the glycolytic gene mutation analysis and drug therapeutic response analysis between the two groups. Finally, the GEO database was employed to validate the impact of glycolysis on TME in patients with EAC.

**Results:** A total of 78 EAC patients with gene expression profiles and clinical information were included for analysis. Functional enrichment results showed that the genes between ESTIMATE ^high^ and ESTIMATE ^low^ groups (*N* = 39, respectively) were strongly related with glycolytic and ATP/ADP metabolic pathways. Patients in the low-risk group had probabilities to survive longer than those in the high-risk group (*p* < 0.001). Glycolytic genes had significant impacts on the components of immune cells in TME, especially on the T-cells and dendritic cells. In the high-risk group, the most common mutant genes were TP53 and TTN, and the most frequent mutation type was missense mutation. Glycolysis significantly influenced drug sensitivity, and high tumor mutation burden (TMB) was associated with better immunotherapeutic response. GEO results confirmed that glycolysis had significant impacts on immune cell contents in TME.

**Conclusion:** We performed a comprehensive study of glycolysis and TME and demonstrated that glycolysis could influence the microenvironment and drug therapeutic response in EAC. Evaluation of the glycolysis pattern could help identify the individualized therapeutic regime.

## Introduction

Esophageal cancer is the eighth most common malignancy and the sixth cause of cancer death globally, which accounts for more than 570,000 new cases and 500,000 deaths annually (Bray et al., 2018; Wang et al., 2018). Esophageal adenocarcinoma (EAC) is the predominant pathological type in western countries, with an increasing proportion from 35 to 61% over the past 30 years (Alsop and Sharma, 2016). The global incidence rate of EAC is approximately 0.7/100,000 person-years, and the 5-year survival rate is merely less than 20%, although multidisciplinary treatments have been applied, including esophagectomy, radiation, and chemotherapy (Arnold et al., 2015; Markar et al., 2017; Smyth et al., 2017; Zhao et al., 2019). Considering the chemotherapeutic resistance, several targeted agents have been applied in patients with EAC, such as imatinib (Mayr et al., 2012). Unfortunately, the efficacy is still not satisfactory. Recently, immunotherapy-targeting PD-1 has revolutionized the therapy in cancer patients. However, not all patients with EAC respond to immunotherapy (Däster et al., 2020). Therefore, there is an urgent necessity to better understand the molecular characteristics and genetic features that could help predict accurate survival and identify suitable patients who will benefit from immunotherapy.

The tumorigenesis and development of EAC is a highly complex biology, involving the tumor cell-intrinsic and cell-extrinsic factors (Quante et al., 2018; Talukdar et al., 2018). Genetic alterations are the primary mechanisms that drive the initiation and progression of EAC, not only conferring tumor cells infinite proliferative abilities but also reprogramming metabolic pathways to adapt to the hostile environment, such as aerobic glycolysis (Hochwald and Zhang, 2017; Talukdar et al., 2018). The seminal discovery of tumor glycolysis has been considered a hallmark of cancer, proposed by Otto Warburg in 1923 (Warburg and Minami, 1923). The glycolytic phenotype renders cancer cells selective advantages by unlimited growth and attenuated apoptosis (Xu et al., 2017). In addition, it is gradually evident that elevated glycolysis is closely related to the immune escape by changing the microenvironment and inhibiting the functions of immune cells (Jiang et al., 2019a). Mounting evidence from cell-based assays has linked glycolysis to TME, and preclinical investigations have demonstrated the effectiveness targeting glycolysis in some cancers (Lim et al., 2017; Kornberg et al., 2018; Jiang et al., 2019a; Jiang et al., 2019b; Kang et al., 2020).

Genetic mutation results in the rewire of the glucose metabolism decreased cancer cell apoptosis and immortal growth. Consequently, these events bring about the component reconstruction in the tumor microenvironment (TME), thus changing the purity of the tumor. Reciprocally, the intimate interactions between glycolytic cells and the extracellular matrix further exacerbate the remodeling of TME, including the stromal and immune cells. It is well accepted that the tumor is highly dependent on TME, which is preponderant on prognosis and impacts the therapeutic efficacy profoundly, such as the immune checkpoint therapy (Wu and Dai, 2017; Alsina et al., 2018; Taube et al., 2018; Hinshaw and Shevde, 2019; Li et al., 2020). However, there are few studies exploring the associations between glycolysis and TME in EAC, and far less is known about how genetic mutations orchestrate the glycolysis under these aberrant TME conditions. Herein, we investigated the effects of glycolysis on immune cells and revealed the genetic mutation diversity. Our study unraveled that glycolysis could influence TME under the driver of genetic mutation and could serve as prognostic biomarkers. Moreover, we constructed the risk score system to predict drug sensitivity and immunotherapeutic response. The results hold great promise in targeting glycolysis and utilizing TME to improve the treatment in patients with EAC.

## Materials and Methods

### Data Acquisition

Gene expression data and clinical information were downloaded from the Cancer Genome Atlas (TCGA) database (https://portal.gdc.cancer.gov/). The mRNA expression profiles were log2 normalized for further analysis. Clinical information included gender, age, stage, survival status, and follow-up time.

### Tumor Microenvironment and Glycolysis

TME is composed of resident stromal cells and infiltrating immune cells (IICs), reflecting tumor purity. With the increase of stromal cells and IICs, the tumor purity becomes lower. The stromal score, immune score, and ESTIMATE score were calculated by applying the ESTIMATE algorithm (Chakraborty and Hossain, 2018). The ESTIMATE score is the comprehensive parameter of the stromal and immune scores. Then, patients with EAC were classified into ESTIMATE ^high^ and ESTIMATE ^low^ groups according to the median of the ESTIMATE score. The differently expressed genes (DEGs) were screened by the weighted gene co-expression network analysis (WGCNA) with the false discovery rate (FDR) ≤0.05 and log2 fold change (log2FC)| >2.

To explore whether glycolysis affects tumor purity, we performed gene set enrichment analysis (GSEA) between the ESTIMATE ^high^ and ESTIMATE ^low^ groups. Five glycolysis-related gene sets, namely, Hallmark, BioCarta, KEGG, GO, and Reactome, were downloaded from the Molecular Signatures Database (http://www.gsea-msigdb.org/gsea/msigdb) and analyzed using the GSEA software (version 4.1.0). The permutation number was set as 1,000 for every phenotype. The gene sets were considered statistically significant when the nominal (NOM) *p*-value ≤0.05, FDR ≤0.05, and normalized enrichment score **|**(NES)**|** >1. Finally, the intersection genes (IGs) from the WGCNA and GSEA gene sets were identified for further analysis.

### Gene Interaction Analysis and Enrichment Analysis

Gene interaction analysis was performed through the “corrplot” package in R software (version 4.1.0). Hub genes were screened with “cytoHubba” in Cytoscape software.

The IGs based on TME and glycolysis were analyzed for Gene Ontology (GO) and Kyoto Encyclopedia of Genes and Genomes (KEGG) by the “clusterProfiler” R package. GO analysis has three functional parts, including the biology process (BP), cellular component (CC), and molecular function (MF).

Next, we performed functional similarity analysis, which was measured through the “GOSemSim” R package (Wang et al., 2007). Functional similarity could be used for the purpose of assessing the intimacy and relationship between each gene and its partners by evaluating the function and location.

### Establishment of Prognostic Signatures

First, univariate Cox regression analysis was used to identify IGs which were related to patients’ overall survival (OS). Then, statistically significant IGs (*p* < 0.05) were enrolled into the multivariate Cox regression. Finally, patients were divided into high- and low-risk groups according to the median of the risk score, in which the risk score was calculated as follows: $\text{risk score}\;=\;{\sum^{\text{​}}}_{n=1}^{j}\;Coefj\;*\;Xj$ , with Coef j indicating the coefficient and Xj representing the relative expression levels of each IG standardized by the z-score.

### TME and Gene Mutation

Next, we selected the prognostic glycolysis-related genes and hub genes to investigate their relations with IICs between the two groups through single-sample gene set enrichment analysis (ssGSEA) using the “GSVA” R package. The effects of OGG on immune cells were assessed using the linear regression.

To analyze why glycolysis affects TME, we calculated the glycolytic gene mutation frequency, variant classification, variant type, and single nucleotide variants (SNVs) between the ESTIMATE ^high^ and ESTIMATE ^low^ groups. Additionally, to fully understand the role of gene mutation in TME, we performed tumor mutation burden (TMB) analyses and explored their relationships with IICs through the simple nucleotide variation data from TCGA and cBioPortal online databases (http://www.cbioportal.org/).

### Drug Sensitivity Analysis and Immunotherapy Response

The drug sensitivity of each patient with EAC was predicted by the Genomics of Drug Sensitivity in Cancer database (GDSC; https://www.cancerrxgene.org/). The half-maximal inhibitory concentration (IC50) was calculated through the “pRRophetic” R package, and the IC50 differences between the high- and low-risk groups were compared (Geeleher et al., 2014).

The response to immunotherapy was estimated using the Tumor Immune Dysfunction and Exclusion website (TIDE; http://tide.dfci.harvard.edu/login/). The TIDE and PDL-1 scores were compared between the high- and low-risk groups.

### External Cohort Validation

**FIGURE 1:**
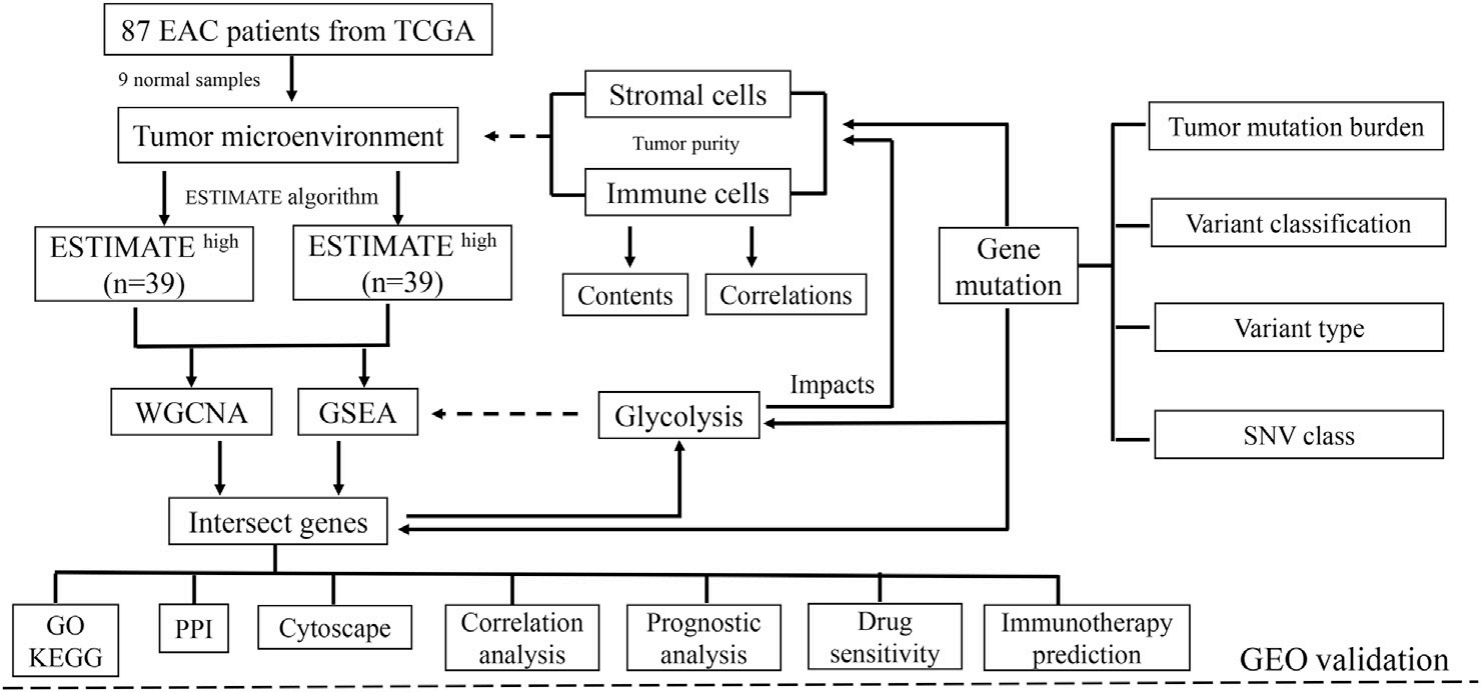
Schematic of the study design. A total of 78 EAC patients were recruited for further study. TME is mainly composed of tumor cells, stromal cells, and immune cells. With higher stromal and immune cells, the tumor purity is low.

The impacts of glycolysis on TME in EAC were validated through the Gene Expression Omnibus (GEO) database (https://www.ncbi.nlm.nih.gov/gds/). The study was considered eligible for external cohort validation according to the following criteria: 1) studies with *Homo sapiens* samples and 2) studies with a sample number more than 50, and 3) studies with detailed experiment information and complete expression profiles. The primary goal of validation was to confirm whether the ESTIMATE algorithm method is suitable for patients with EAC, and the secondary goals were to determine whether glycolysis could influence the components of the microenvironment and affect drug sensitivity. The overall design of this study is shown in Figure 1.

### Statistical Analysis

All statistical analyses were performed by the R software (version 4.1.0). The DEG analysis between the ESTIMATE ^high^ and ESTIMATE ^low^ groups was carried out by applying the unpaired *t-*test. Cox regression analysis was used to determine the prognostic factors. Kaplan–Meier (K-M) curves and log-rank tests were utilized to assess the prognostic outcome. The Mann–Whitney *U* test was used to compare the immune score, immune cell infiltrations, and immune signatures. Spearman’s correlation analysis was used to evaluate the interactions. *p* < 0.05 was considered significant.

## Results

### Identification of Tumor Purity and Glycolysis

**FIGURE 2:**
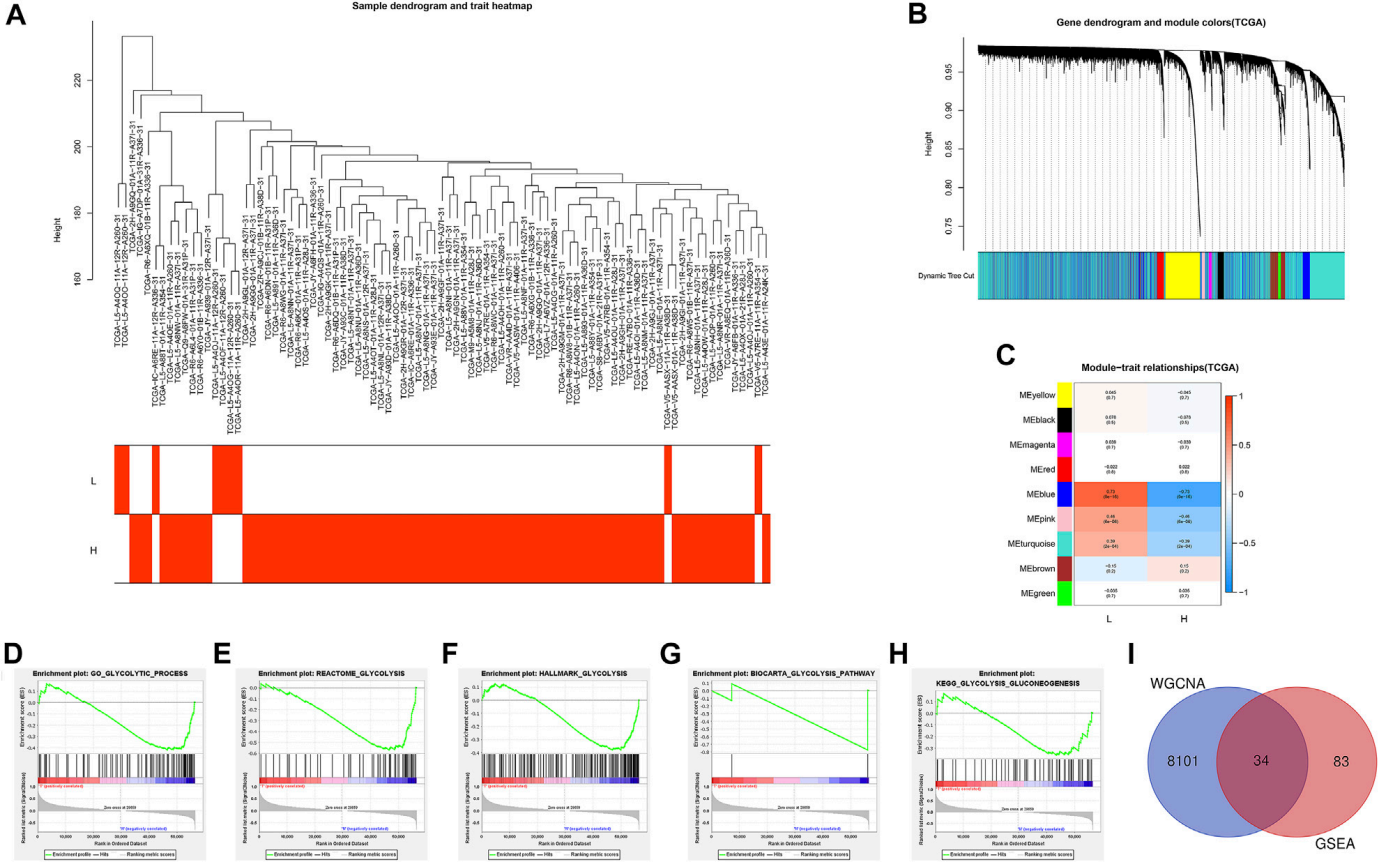
Identification of the intersection genes. **(A)** 78 EAC sample clustering. All samples were clustered, and difference analysis was performed. **(B)** Gene dendrogram and dynamic tree cuts. Each color represents a module, and genes with similar expression patterns will be classified into the same module. **(C)** Module-trait relationships. Every row represents a module eigengene (ME). The red module corresponds to the significant positive correlation, and blue corresponds to the significant negative correlation. **(D)** GO glycolytic process gene set. **(E)** Reactome glycolysis gene set. **(F)** Hallmark glycolysis gene set. **(G)** Biocarta glycolysis pathway. **(H)** KEGG glycolysis gluconeogenesis. **(I)** Venn diagram shows the 34 IGs (intersection genes from WGCNA and GSEA). L: ESTIMATE ^low^; H: ESTIMATE ^high^.

A total of 87 EAC samples and gene expression data were available from the TCGA database, including 9 normal and 78 EAC cases. Based on the ESTIMATE algorithm, the stromal score ranged from –2,315.387 to 1903.167 and the immune score ranged from –1,224.491 to 3,362.338. The range of the comprehensive ESTIMATE score was from –3,375.446 to 5,265.505 (Supplementary Table S1). According to the median of the ESTIMATE score, 78 patients with EAC were categorized into the ESTIMATE ^high^ and ESTIMATE ^low^ groups (39 cases, respectively). There are 8,135 DEGs between two groups according to WGCNA results (Figures 2A–C) (Supplementary Table S2).

Then, GSEA was conducted to assess the glycolytic differences between the ESTIMATE ^high^ and ESTIMATE ^low^ groups. The results showed that the GO glycolytic process (NES = −1.54, NOM *p* = 0.050, FDR = 0.050) and Reactome glycolysis (NES = −1.83, NOM *p* = 0.008, FDR = 0.008) were significantly enriched in ESTIMATE ^high^ group patients (Figures 2D,E). There were no significant enrichments in the Hallmark (NES = −1.44, NOM *p* = 0.067, FDR = 0.067), BioCarta (NES = −1.23, NOM *p* = 0.227, FDR = 0.227), and KEGG (NES = −1.20, NOM *p* = 0.228, FDR = 0.228) pathways (Figures 2F–H). There were 117 DEGs between GO and Reactome glycolysis gene sets. After screening, a total of 34 IGs were selected from WGCNA and GSEA for further analysis (Table 1) (Figure 2I). The details about IGs in each EAC sample are shown in Supplementary Table S3.

**TABLE 1:** Significant IG expression levels in the ESTIMATE ^low^ and ESTIMATE ^high^ tissues.

Gene	ESTIMATE ^low^	ESTIMATE ^high^	logFC	*p*	FDR
GAPDH	693.986	509.158	−0.447	0.002	0.011
NUP133	9.617	8.402	−0.195	0.035	0.118
GCK	0.077	0.232	1.595	0.000	0.001
HTR2A	0.037	0.323	3.116	0.000	0.000
NUP205	15.387	12.149	−0.341	0.037	0.122
NUP43	11.641	9.217	−0.337	0.000	0.004
SEH1L	7.027	5.367	−0.389	0.023	0.086
INSR	11.440	15.775	0.464	0.003	0.017
BPGM	5.508	7.941	0.528	0.000	0.000
PRKACB	4.543	6.572	0.533	0.010	0.045
CBFA2T3	0.390	1.505	1.947	0.000	0.000
NUP214	9.564	8.187	−0.224	0.015	0.062
NDC1	16.439	12.273	−0.422	0.001	0.006
HK3	0.409	1.216	1.572	0.000	0.000
NUP62	14.909	12.765	−0.224	0.027	0.096
EIF6	105.279	83.551	−0.333	0.022	0.084
PRXL2C	4.076	5.085	0.319	0.020	0.080
DDIT4L	0.098	0.215	1.134	0.000	0.001
ZBTB20	1.195	1.939	0.699	0.001	0.006
ENO1	227.228	180.617	−0.331	0.006	0.031
MLXIPL	7.779	4.578	−0.765	0.001	0.005
PRKAG1	9.508	8.284	−0.199	0.022	0.084
P2RX7	0.417	1.300	1.641	0.000	0.000
RAE1	10.815	8.043	−0.427	0.002	0.012
GPI	48.943	35.859	−0.449	0.005	0.026
NUP188	19.729	17.185	−0.199	0.037	0.122
HIF1A	49.095	66.493	0.438	0.002	0.014
HKDC1	18.783	12.578	−0.579	0.039	0.127
NUP37	6.737	5.076	−0.408	0.002	0.013
HK1	16.837	20.341	0.273	0.045	0.140
IGF1	0.090	0.414	2.196	0.000	0.000
TPI1	142.420	116.178	−0.294	0.025	0.091
ENO3	1.130	0.929	−0.281	0.022	0.084
NUP88	10.051	8.744	−0.201	0.045	0.140

LogFC: log fold change; FDR: false discovery rate.

### Gene Interaction Networks and Functional Enrichment Analysis

**FIGURE 3:**
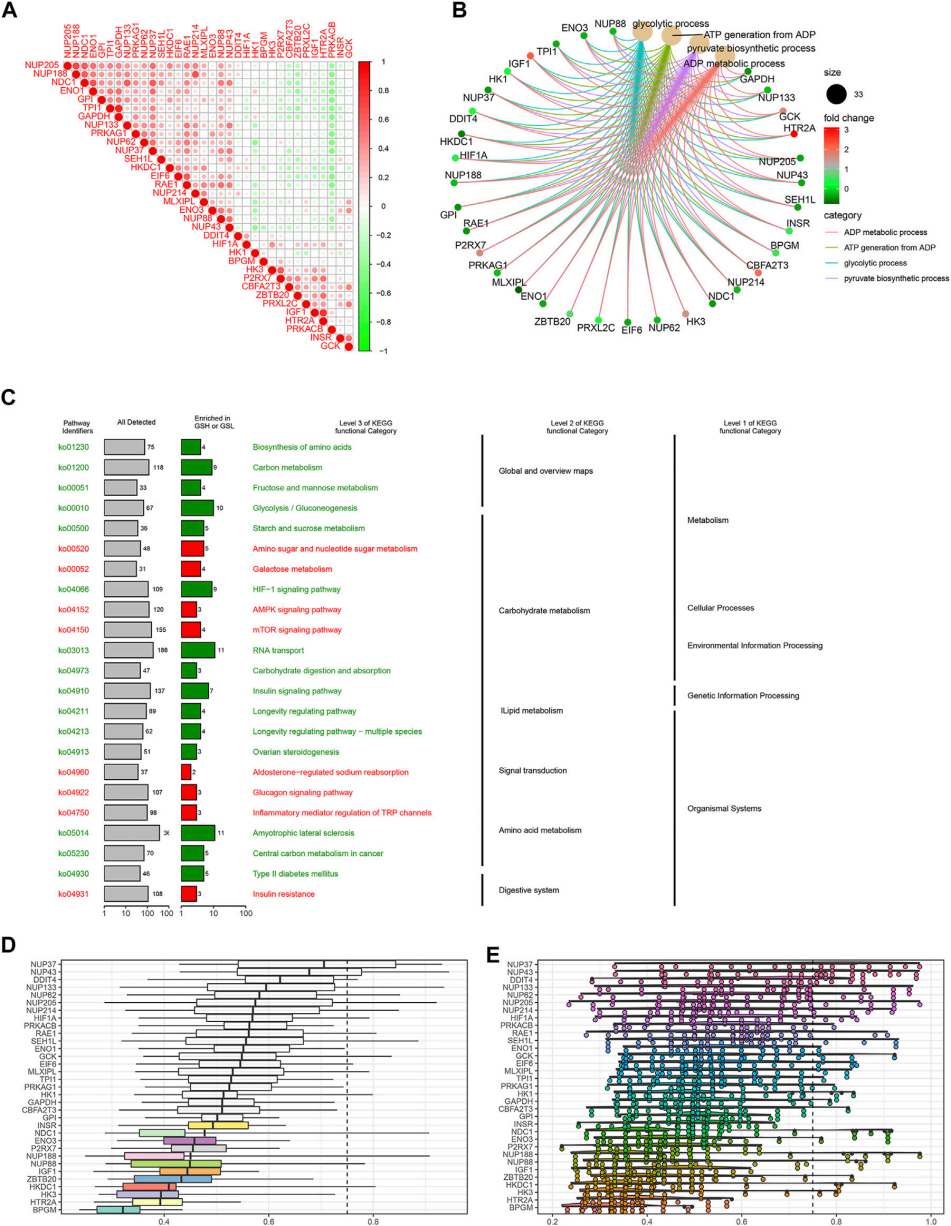
Gene interaction analysis and functional enrichment. **(A)** Gene interaction network. Red represents positive correlation, while negative correlation is represented in green. **(B)** GO enrichment analysis, including the biological process (BP), cellular component (CC), and molecular function (MF). Every term shows top 10 pathways. **(C)** Kyoto Encyclopedia of Genes and Genomes (KEGG) pathways enriched in the IGs. IG network analysis. **(D)** Summary of OGG similarities. The boxes indicated the middle 50% of the similarities, and the upper and lower boundaries show the 75th and 25th percentiles, respectively. **(E)** Raincloud plots of OGG. Data are expressed as the mean and standard error. Each dot represents the single gene. The dashed line represents the cutoff value (0.75).

To explore the correlation between the IGs, we calculated their coefficients. Gene interaction analysis showed that GAPDH and TPI1 had the strongest positive correlation (coef = 0.81), whereas PRKACB and RAE1 had the strongest negative correlation (coef = −0.48) (Figure 3A). To explore the IG functions, we performed GO and KEGG enrichment analyses using R packages. GO results showed that IGs were significantly enriched in the glycolytic and ATP/ADP metabolic pathways. In addition, nuclear-, glucose-, and carbohydrate-related activities were closely associated with CC and MF terms (Figure 3B). KEGG results demonstrated that carbon, gluconeogenesis, and HIF−1 signaling pathways were enriched (Figure 3C).

Based on the GO analysis and semantic similarities, we ranked the genes by average functional similarities between IGs and their partners, with the cutoff value at 0.75. The box plots and raincloud plots are demonstrated in Figures 3D,E. From the pictures, we can clearly see that NUP43, NUP37, and DDIT4 had strong similarities and weak correlation with BPGM.

### Prognostic IG Signatures

**FIGURE 4:**
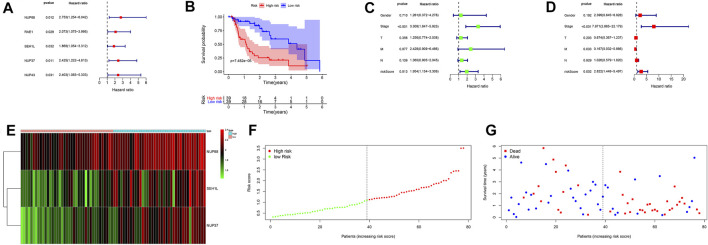
Prognostic signatures of OS in EAC patients. **(A)** Forest plot of univariate Cox regression analysis based on IGs. **(B)** K-M survival plot of high- and low-risk score patients. **(C)** Forest plot of univariate Cox regression analysis based on the clinical information and risk score. **(D)** Forest plot of multivariate Cox regression analysis based on the clinical information and risk score. **(E)** Heatmap of NUP88, NUP37, and SEH1L in EAC patients. Green represents low gene expression, and red represents high expression. **(F)** Risk score curve of high- and low-risk score patients. The dotted line represents every individual, and patients are categorized into low-risk and high-risk groups at the inflection point. **(G)** Survival status and time distributed by the risk score. The red dot represents the dead, and blue represents the living. With an increase in time, more and more patients died. T: tumor; M: metastasis; N: lymph node.

Univariate Cox regression analysis revealed that NUP88, RAE1, SEH1L, NUP37, and NUP43 were significantly associated with patients’ OS (all *p* < 0.05) (Figure 4A). After multivariate Cox regression analysis, three IGs (NUP88, SEH1L, and NUP37) were used to develop the risk score based on the following formula: risk score = 0.637 * expression of NUP88 + 0.494 * expression of SEH1L + 0.657 * expression of NUP37. Also, the three genes were all risk genes with hazard ratio (HR) > 1. A total of 78 patients with EAC were classified into low- and high-risk groups according to the median risk score (*n* = 39). The K-M survival plot showed that patients in the low-risk group had significant probabilities to survive longer than those in the high-risk group (median time = 1.75 vs 0.745 years, *p* < 0.001) (Figure 4B).

In order to evaluate the prognostic values of clinical information in OS, we integrated the patients’ clinical features with IGs. Univariate Cox regression analysis showed that the tumor stage (HR = 3.308, *p* < 0.001) and risk score (HR = 1.954, *p* = 0.013) were significantly associated with OS (Figure 4C). Multivariate Cox regression analysis results demonstrated that tumor stage (HR = 7.971, *p* < 0.001), metastasis (HR = 0.167, *p* = 0.033), and risk score (HR = 2.822, *p* = 0.002) were independent risk factors for OS (Figure 4D). In addition, the distributions of each patient and their survival statuses are shown in Figures 4E–G. We can clearly see that patients in the low-risk group had a better prognosis than those in the high-risk group.

### Effect of Glycolytic Genes on IICs

**FIGURE 5:**
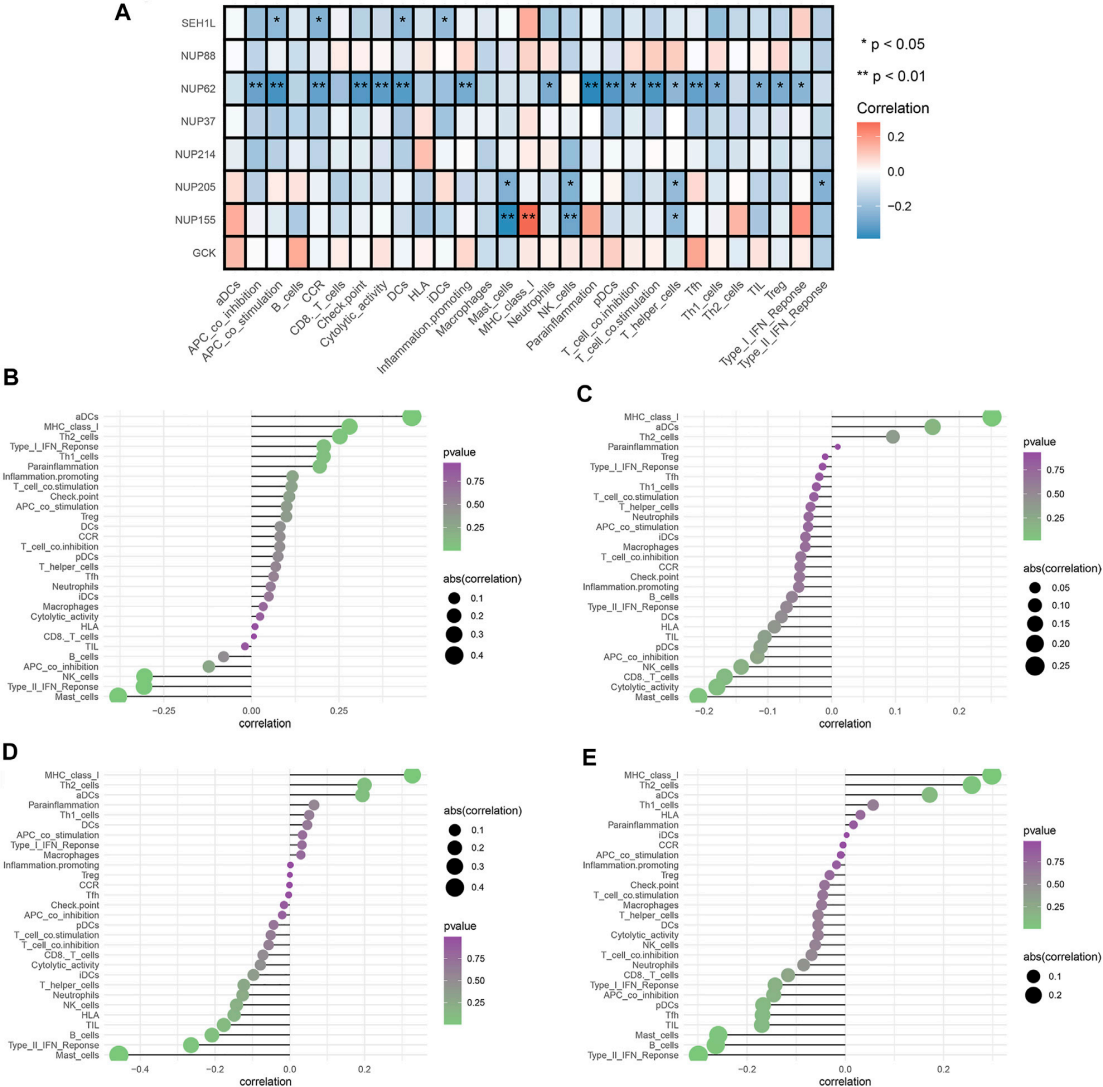
Analysis of glycolytic gene effects on immune signatures. **(A)** Heatmap demonstrating the correlation between 8 genes and the ssGSEA scores of 29 immune signatures. **(B)** NUP62 and immune signatures. **(C)** NUP155 and immune signatures. **(D)** NUP205 and immune signatures. **(E)** SEH1L and immune signatures. Spearman’s correlation analysis was used to evaluate the relations with *p* < 0.05.

We selected three prognostic genes and five hub genes (NUP88, SEH1L, NUP37, GCK, NUP62, NUP155, NUP205, and NUP214) to assess whether glycolysis affects the IICs in TME. The results demonstrated that NUP62, NUP155, NUP205, and SEH1L had significant impacts on the IIC expression level, especially on the T-cells and mast cells (all *p* < 0.05). The details are shown in Figure 5A.

To fully explore the relationships between these genes and IICs, we performed Spearman correlation analysis by using the “Limma” package. The results showed that NUP62 was strongly associated with T-cells, dendritic cells, and antigen-presenting cells (all *p* < 0.05) (Figure 5B). NUP155 had a close relationship with T-cells, mast cells, and MHC class I activity (all *p* < 0.05) (Figure 5C). NUP205 showed close relationships with T-cells, mast cells, and type I IFN response (all *p* < 0.05) (Figure 5D). SEH1L exhibited significant associations with dendritic cells, antigen-presenting cells, and chemokine receptors (CCR) (all *p* < 0.05) (Figure 5E). Collectively, these findings suggested that the IGs had profound effects on immune cells and immunological functions.

### Genetic Mutation and the Tumor Microenvironment

**FIGURE 6:**
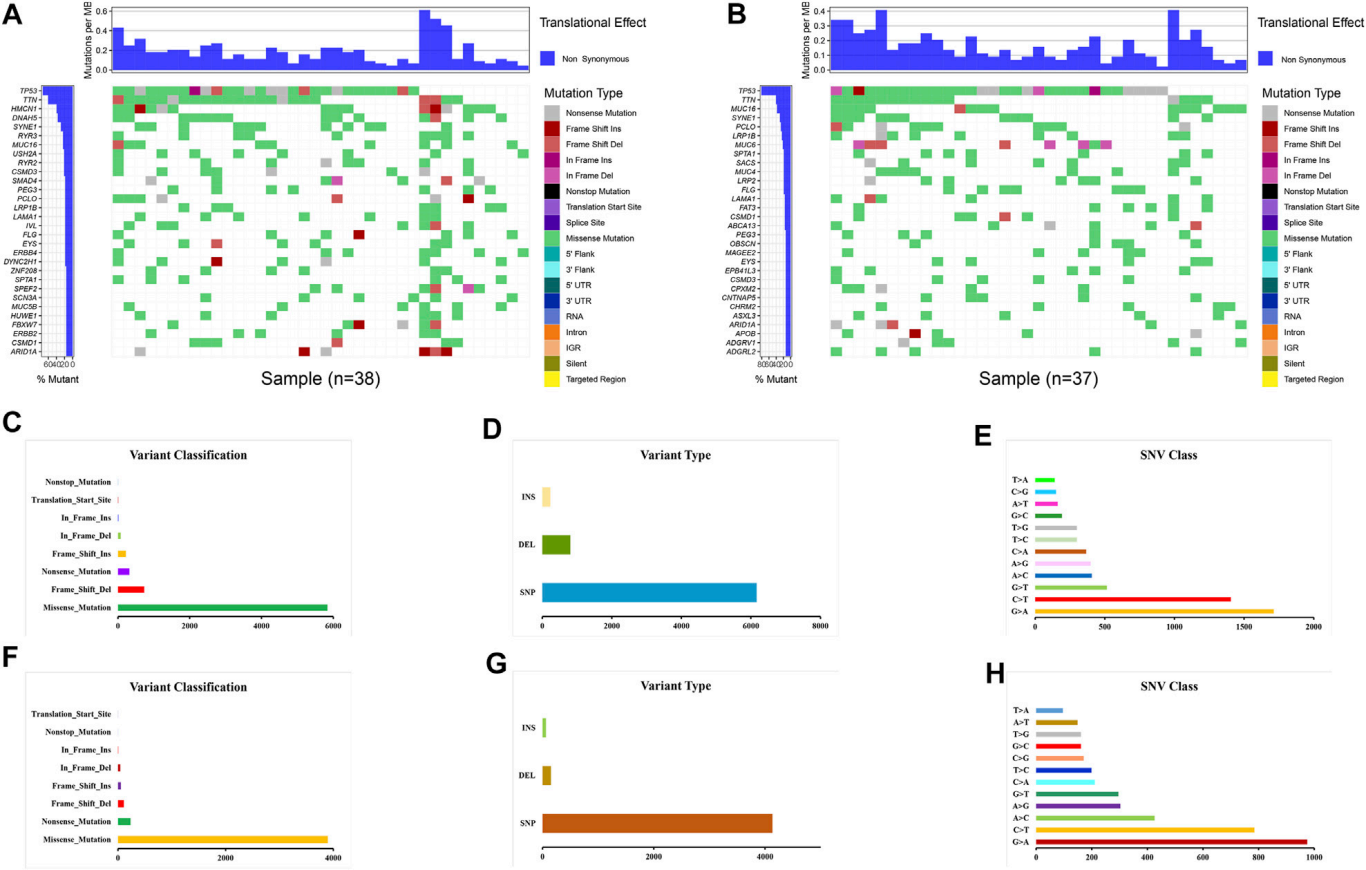
Landscape of gene mutations in EAC patients. **(A)** Waterfall plot of mutational genes in the ESTIMATE ^high^ group. **(B)** Waterfall plot of mutational genes in the ESTIMATE ^low^ group. The left panel shows the gene mutational frequencies, and the right panel represents the mutational type. **(C)** Variant classification and frequency of gene mutations in the ESTIMATE ^high^ group. **(D)** Variant type in the ESTIMATE ^high^ group. **(E)** Frequency of SNV classes in the ESTIMATE ^high^ group. **(F)** Variant classification and frequency of gene mutations in the ESTIMATE ^low^ group. **(G)** Variant type in the ESTIMATE ^low^ group. **(H)** Frequency of SNV classes in the ESTIMATE ^low^ group.

Gene mutations were analyzed for further investigation into the mechanisms that TME is affected by gene alteration. The genetic mutations are significantly different between the ESTIMATE ^high^ and ESTIMATE ^low^ groups. In the ESTIMATE ^high^ group, the five most common mutant genes were TP53, TTN, HMCN1, DNAH5, and SYNE1, and the most common mutational type was missense mutation (Figures 6A,C). In the ESTIMATE ^low^ group, the most common mutant genes were TP53, TTN, MUC16, SYNE1, and PCLO. The most common mutational type was also missense mutation (Figures 6B,F). The frequencies of missense mutation were significantly lower than that in the ESTIMATE ^high^ group, indicating that the glycolytic level and tumor purity were different from those of the ESTIMATE ^high^ group (Figures 6A,B). Single-nucleotide polymorphism (SNP) had the highest frequency in the variant type (Figures 6D,G). G > A was the most frequent type in the SNV class (Figures 6E,H). The results imply that these mutant genes drive a higher glycolytic level, consequently changing the tumor purity in the microenvironment.

**FIGURE 7:**
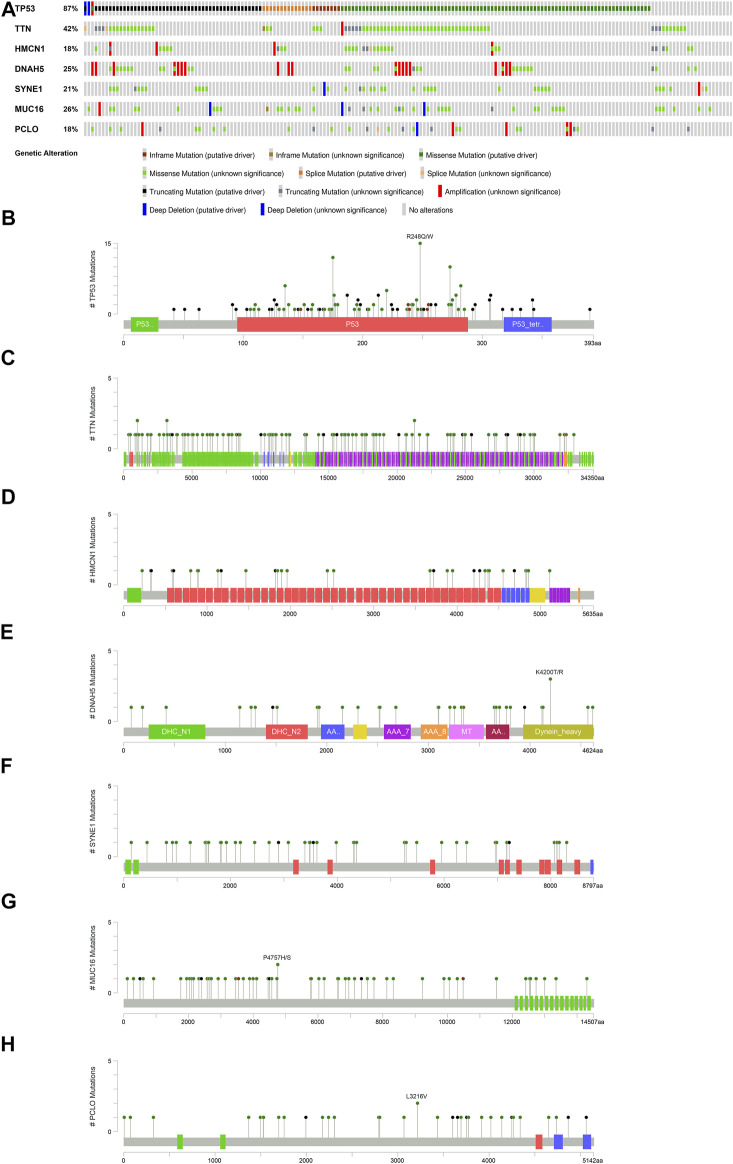
Overview of the seven most common mutant genes in EAC patients. **(A)** Proportion and mutation type of genes. Different color bars represent different mutational types. **(B)** TP53-specific mutation site. **(C)** TTN-specific mutation site. **(D)** HMCN1-specific mutation site. **(E)** DNAH5-specific mutation site. **(F)** SYNE1-specific mutation site. **(G)** MUC16-specific mutation site. **(H)** PCLO-specific mutation site.

To broaden the understanding of the glycolytic gene mutations, the cBioPortal database was applied to validate these findings. We selected the most common glycolytic genes (TP53, TTN, HMCN1, DNAH5, SYNE1, MUC16, and PCLO) for further verification. Consistent with the above findings, the results from the cBioPortal database showed that TP53, TTN, and MUC16 possessed the highest mutation frequencies (87, 42, and 26%, respectively), and missense mutation was the commonest type (Figure 7).

### Tumor Mutation Burden Analysis and Immunotherapy Response

**FIGURE 8:**
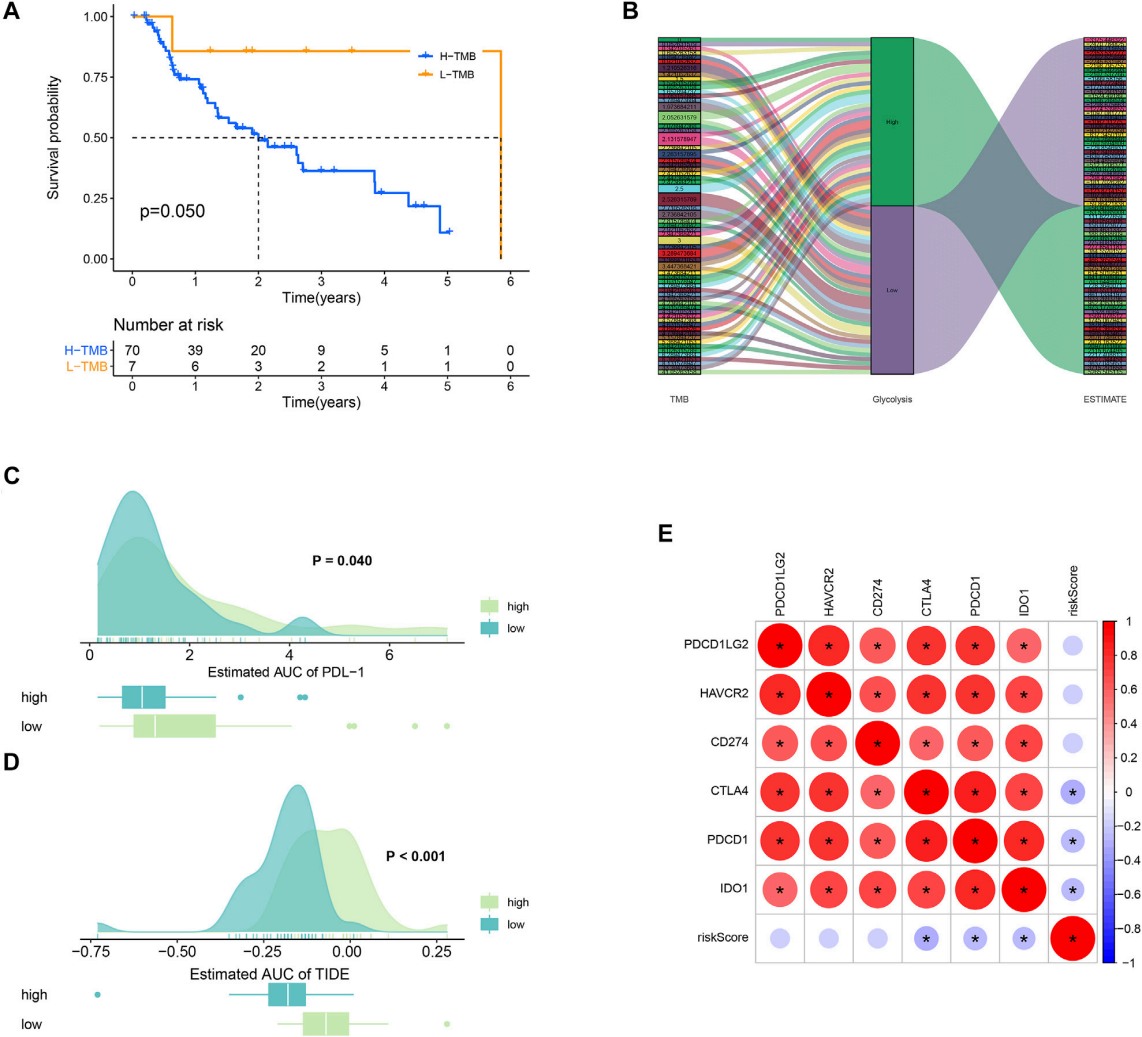
TMB and immunotherapy response. **(A)** K-M survival plot. Patients with low TMB have significant probabilities to survive longer than those with high TMB. **(B)** Sankey diagram showing the relations between TMB, glycolysis, and the ESTIMATE score. **(C)** PD-L1 score comparison between high- and low-TMB groups. **(D)** TIDE algorithm analysis showed that patients with high TMB had better immunotherapeutic response than those with low TMB. **(E)** Correlations plots between the immune checkpoints.

TMB refers to the total number of mutations per mega base in tumor tissues. By analyzing the SNP data downloaded from TCGA, we calculated the TMB frequency in each patient with EAC. The range of TMB is from 0.053 to 41.053 in EAC. Moreover, we further analyzed the effect of TMB on survival in patients with EAC. A total of 78 patients were classified into high- and low-TMB groups according to the median TMB. As shown in the K-M curves, patients in the high-TMB group had significantly higher mortality than those in the low-TMB group (*p* = 0.05) (Figure 8A). To exhibit the relationships between TMB, glycolysis, and TME (ESTIMATE score), we applied the Sankey diagram to visualize their correlations with the “ggalluvial” package in R. The result is shown in Figure 8B.

Low TMB usually implies poor response for immunotherapy (Chan et al., 2019). To explore whether TMB will influence the immunotherapy response, we compared the PDL-1 and TIDE scores between high- and low-TMB groups. As a result, patients in the high-TMB group significantly responded to anti–PDL-1 therapy (*p* = 0.040) (Figure 8C). In Figure 8D, patients with high TMB had a higher TIDE score than those with low TMB (*p* = 0.000). In addition, the correlations between the immune checkpoints are also explored in Figure 8E.

### Drug Sensitivity Analysis

**FIGURE 9:**
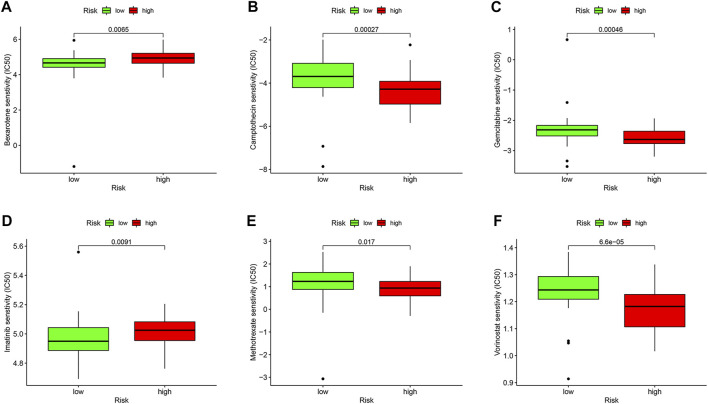
Drug sensitivity analysis in the TCGA database. Box plots demonstrate the estimated IC50 values of bexarotene **(A)**, camptothecin **(B)**, gemcitabine **(C)**, imatinib **(D)**, methotrexate **(E),** and vorinostat **(F)**. The lower the IC50 value, the higher is the sensitivity to the drug.

We compared the IC50 differences of chemotherapeutic and targeted drugs between high- and low-risk score groups, including bexarotene (Figure 9A), camptothecin (Figure 9B), gemcitabine (Figure 9C), imatinib (Figure 9D), methotrexate (Figure 9E), and vorinostat (Figure 9F). The results demonstrated that there were higher IC50 levels of bexarotene and imatinib in the high-risk score group, which indicated that patients with a low-risk score were more sensitive to the two drugs. Oppositely, the IC50 levels of camptothecin, gemcitabine, methotrexate, and vorinostat were higher in the low-risk score group, implying that patients in the high-risk score group were more sensitive to the four drugs.

### Validation of Glycolysis and Its Impact on TME

For external cohort validation, GSE12898 was employed, which consisted of 75 EAC samples. Consistent with the classification result of the ESTIMATE algorithm in TCGA, this method successfully divided 75 patients with EAC into the ESTIMATE ^high^ and ESTIMATE ^low^ groups (*n* = 38 and 37, respectively). There were 3,428 genes between the two groups, including 52 significantly different glycolysis-related genes (Supplementary Table S4). The correlation analysis showed that 28 glycolytic genes had significant impacts on immune cells in the microenvironment, and the majority were B-cell and T-cell subtypes. The details are shown in Table 2.

**TABLE 2:** Impacts of glycolytic genes on immune cells in TME.

Type	B n	B m	M2	Mono	CD4 a	CD4 r	CD4 n	Tfh	NK r	Neu	DC a	γδT
ADPGK	0.016*	0.490	0.037*	0.199	0.482	0.728	0.922	0.881	0.336	0.934	0.892	0.768
ALG1	0.037*	0.119	0.254	0.723	0.238	0.376	0.718	0.385	0.762	0.269	0.765	0.670
CACNA1H	0.457	0.535	0.031*	0.805	0.157	0.166	0.372	0.673	0.846	0.676	0.366	0.409
CHST12	0.230	0.739	0.201	0.010*	0.567	0.367	0.713	0.927	0.250	0.159	0.390	0.713
COL5A1	0.080	0.637	0.024*	0.018*	0.129	0.437	0.718	0.653	0.680	0.472	0.514	0.158
CXCR4	0.067	0.856	0.013*	0.339	0.298	0.769	0.718	0.881	0.260	0.678	0.828	0.768
DCN	0.037*	0.446	0.330	0.076	0.312	0.538 0.870	0.976	0.196	0.507	0.532	0.450	
DPYSL4	0.259	0.488	0.556	0.527	0.009*	0.727	0.137	0.976	0.361	0.738	0.702	0.120
DSC2	0.123	0.913	0.889	0.178	0.734	0.767	0.197	0.047*	0.719	0.330	0.460	0.155
FUT8	0.007*	0.490	0.126	0.287	0.555	0.320	0.922	0.590	0.196	0.934	0.765	0.974
GALK2	0.012*	0.075	0.303	0.891	0.216	0.689	0.922	0.590	0.492	0.580	0.807	0.870
GPC3	0.039*	0.053	0.300	0.094	0.713	0.267	0.869	0.470	0.455	0.559	1.000	0.120
GPC4	0.016*	0.119	0.277	0.643	0.312	0.347	0.718	0.385	0.237	0.934	0.496	0.577
GUSB	0.132	0.537	0.079	0.643	0.238	0.470	0.158	0.905	0.045*	0.619	0.683	0.224
HSPA5	0.037*	0.637	0.070	0.219	0.978	0.728	0.718	1.000	0.336	0.580	0.807	0.870
KDELR3	0.007*	0.490	0.126	0.287	0.555	0.320	0.922	0.590	0.196	0.934	0.765	0.974
NDUFV3	0.642	0.537	0.277	0.339	0.030*	0.574	0.158	0.255	0.051	0.333	0.978	0.818
NT5E	0.396	0.690	0.015*	0.076	0.555	0.689	0.533	0.491	0.309	0.176	0.957	0.718
NUP210	0.659	0.742	0.110	0.051	0.224	0.979	0.197	0.195	0.489	0.019*	0.956	0.974
PFKFB3	0.508	0.116	0.123	0.564	1.000	0.025*	0.447	0.880	0.934	1.000	0.035*	0.716
PLOD1	0.539	0.970	0.094	0.522	0.197	0.892	0.366	0.329	0.978	0.866	0.868	0.048*
PLOD2	0.870	0.046*	0.597	0.429	0.757	0.437	0.039*	0.072	0.309	0.825	0.870	0.158
SDC2	0.013*	0.361	0.153	0.197	0.224	0.936	0.372	0.833	0.978	0.867	0.622	0.120
STC1	0.338	0.690	0.079	0.604	0.933	0.503	0.224	0.454	0.022*	0.868	0.663	0.670
TGFBI	0.639	0.535	0.053	0.045*	0.045*	0.851	0.372	0.292	0.306	0.303	0.239	0.530
TPST1	0.934	0.635	0.077	0.011*	1.000	0.687	0.574	0.382	0.391	0.182	0.239	0.221
VCAN	0.218	0.856	0.021*	0.067	0.072	0.376	0.718	0.436	0.284	0.376	0.913	0.251
ZBTB20	0.119	0.537	0.172	0.643	0.086	0.810	0.158	0.811	0.039	0.619	0.978	0.577

B n: naïve B-cells; B m: memory B-cells; M2: M2 macrophages; Mono: monocytes; CD4 a: memory-activated CD4 T-cells; CD4 r: memory resting CD4 T-cells; CD4 n: naïve CD4 T-cells; Tfh: follicular helper T-cells; NK r: resting NK cells; Neu: neutrophils; DC a: activated dendritic cells; γδT: gamma delta T-cells.

*: *p*<0.05.

**FIGURE 10:**
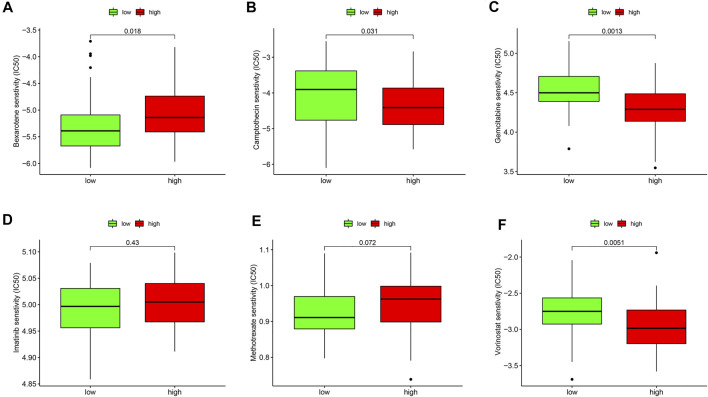
Validation of drug sensitivity analysis in the GEO database. Box plots demonstrate the estimated IC50 values of bexarotene **(A)**, camptothecin **(B)**, gemcitabine **(C)**, imatinib **(D)**, methotrexate **(E),** and vorinostat **(F)**. The lower the IC50 value, the higher is the sensitivity to the drug.

Furthermore, the drug sensitivities were also analyzed between the ESTIMATE ^high^ and ESTIMATE ^low^ groups. The results showed that the IC50 level of bexarotene (Figure 10A) was higher in the ESTIMATE ^high^ group. However, the IC50 levels of camptothecin (Figure 10B), gemcitabine (Figure 10C), and vorinostat (Figure 10F) were lower in the ESTIMATE ^high^ group, implying that the patients in the ESTIMATE ^high^ group were more sensitive to the four drugs. There were no significant differences regarding IC50 in imatinib (Figure 10D) and methotrexate (Figure 10E). Taken together, glycolysis directly changed TME and indirectly influenced drug sensitivity.

## Discussion

The abilities of cancer cells to switch metabolisms and evade the immunity system in TME are well-documented characteristics in tumors. Elevated glycolysis is commonly observed in cancer progression and is associated with significant disruptions of a previous finely tuned microenvironment (Chang et al., 2015; Jiang et al., 2019a). As the tumors develop, they constantly interact with neighboring cells, such as stromal cells and immune cells, under the driver of genetic mutations, thus altering their phenotypes and functions (Butturini et al., 2019). In the context of these intricate crosstalks between malignant cells and non-malignant cells, the impacts of increased glycolysis and a dysregulated TME on immune response and effective therapy are of vital importance (Roma-Rodrigues et al., 2019). Although the research studies focusing on the tumor glycolysis and TME have exploded exponentially in recent years, the underlying mechanisms of how they act both independently and synergistically still remain elusive. In this study, we explored systematically the links between glycolysis and TME in patients with EAC as well as the relations with genetic mutations. In the present study, we found that the glycolytic level was higher in the ESTIMATE ^high^ group, reasonably reflecting the fact that glycolysis may change the tumor purity. By constructing the predictive survival model based on IG signatures, we discovered that three genes (NUP88, SEH1L, and NUP37) may serve as independent prognostic biomarkers for OS in EAC. In addition, we revealed that gene mutation types and frequencies were distinct between the different ESTIMATE score groups, lending us a hypothesis that genetic alteration may drive TME changes. In addition, our data suggested that glycolysis could influence drug sensitivity and immunotherapeutic response.

Several studies have confirmed the close relationships between glycolysis and EAC (Lynam-Lennon et al., 2014; Harada et al., 2020; Kang et al., 2020). Consistent with these findings, our functional enrichment analysis results showed that IGs were strongly enriched in the glycolytic processes, ATP generation, the HIF−1 signal pathway, RNA activities, and so on. The presence of aerobic glycolysis under normal conditions efficiently promotes tumor cell growth by the following mechanisms: 1) the rate of glycolysis is accelerated at 100 times compared to oxidative phosphorylation to compensate for the mathematical disadvantage in terms of net ATP production (glucose oxidative phosphorylation although mitochondria generate 18 times ATP compared to glycolysis) (Pfeiffer et al., 2001); 2) glycolysis provides sufficient and essential intermediates, such as NADPH and ribose-5-phosphate, which are indispensable for biosynthesis to meet avid proliferative requirements (Boroughs and DeBerardinis, 2015); 3) lactate, the obligatory product derived from glycolysis, could activate the HIF−1 signal pathway to induce vascular endothelial growth factor (VEGF) expression to stimulate angiogenesis in the microenvironment (Sonveaux et al., 2012). In addition, lactate is crucial to reorganize the tumor physical architectures in TME, and the accumulation of extracellular lactate is detrimental for normal healthy cells, such as immune cells (Romero-Garcia et al., 2016; Cassim and Pouyssegur, 2019). The study about how RNA interacts precisely with glycolysis is still in its infancy. However, Hua Q et al. gave us a hint that long non-coding RNA may promote glycolysis by sponging miRNA (Hua et al., 2019). Hence, it is reasonable to speculate that certain mutational genes regulated and modified by RNA may be involved in the glycolysis in EAC (Hochwald and Zhang, 2017).

The prognostic signature of glycolysis in EAC was established based on three genes (NUP88, SEH1L, and NUP37). NUP88, located at chromosome 17p13, encodes Nup88 protein (Zhao et al., 2012). Nup88 is a nucleoporin comprising nuclear pore complexes (NPCs) and plays critical roles in maintaining the spindle stability and preventing aneuploidy formation during mitosis (Hashizume et al., 2010). Unanimously, the GO results in our study also illustrated that glycolytic genes had intimacy with the nuclear pore, emphasizing the importance of nuclear proteins in glycolysis. The Nup88 overexpression is highly associated with tumor development and decreased survival, suggesting that NUP88 acts as an oncogene (Martínez et al., 1999; Naylor et al., 2016). In line with these studies, our results also proved that NUP88 was a risk factor for OS (HR > 1). Another prognostic gene is NUP37, which shares similarities with NUP88 and is also a member of NPC. It exerts primary functions of sustaining NPC integrity and modulates the cell cycle (Chen et al., 2019). Previous studies showed that the elevated expression of NUP37 is associated with worsened survival rates in liver cancer (Uhlen et al., 2017). In addition, a latest study by Huang L et al. demonstrates that NUP37 silencing induces inhibition of lung cancer cell proliferation (Huang et al., 2020). These findings were in agreement with our results, pointing out that NUP37 played an oncogenic role in OS (HR > 1). Nonetheless, the function of NUP37 in EAC has never been explored and needs further experiments to confirm *in vitro* and *in vivo*. SEH1L, also known as Seh1, is a part of NPC as well. However, the field is still in its infancy, and only a handful of animal models have been developed to investigate the role of SEH1L. Studies have shown that Seh1 could promote oligodendrocyte differentiation (Liu et al., 2019; Raices and D’Angelo, 2019). However, little is known about how it works in EAC. Undeniably, more research studies are warranted to understand the specific effects of SEH1L on EAC.

The notion that glycolysis has profound impacts on immune cells in TME is well recognized. The investigation linking metabolic demands and immune cells was first documented in neutrophils and macrophages (Alonso and Nungester, 1956; Newsholme et al., 1986). Immune cells hold a resemblance with tumor cells to engage glycolysis, which require rapid energy sources to produce immune-mediators for migration and phagocytosis. Our study demonstrated that NUP88 had significant correlation with B-cells and mast cells (all *p* < 0.05) and NUP37 had positive correlation with T-cells and negative correlation with dendritic cells (DCs) and macrophages (all *p* < 0.05). These findings enforced the concept that glycolysis contributes to dramatic alterations of immune cells in TME, hence influencing the immune response and immune-based treatments. Glycolytic tumor cells compete with T-cells for glucose and impair T-cell activation (Peng et al., 2016). In addition, IFN-γ secreted by Th1 cells is sensitive and is unstable to lactate. Noteworthily, this could polarize the T-cells toward differentiating into the Th2 subpopulation that favors tumor progression by inhibiting the antitumor effect of M1 macrophages (Kareva and Hahnfeldt, 2013). Paradoxically, direct evidence from *in vivo* experiments supports that glycolysis could promote Th1 cell differentiation through an epigenetic mechanism (Peng et al., 2016). This calls for further studies aiming to shed light on the characteristics of glycolysis on T-cells. The divergent effects of glycolysis on DC are also observed. Glycolysis produces excessive lactate and lowers the pH in TME. Lactate together with decreased pH suppresses DC differentiation and consequently abolishes the antigen-presenting functions to T-cells (Harmon et al., 2020). However, inhibition of glycolysis also blocks DC maturation through HIF−1α. This highlights the multiple roles of DC in glycolysis and needs to be carefully interpreted in a context-dependent manner.

Gene mutations facilitate tumor cell metabolic plasticity to create favorable microenvironments beneficial to uncontrolled proliferation. Deepening our knowledge about the differences of gene alterations between different TMEs may allow for the development of therapeutic strategies. Spurred by this promising target, we explored upon this issue and showed that ESTIMATE ^high^ and ESTIMATE ^low^ groups manifested different gene mutation profiles, in which TP53 and TTN were the most prevalent mutant genes. Moreover, the most common type is missense mutation. Mutant TP53 endows tumor cells with adaptabilities to cope with the harsh microenvironment by providing adequate nutrients, thereby escaping from antitumor immune attack. Experimental mice models in the study by Basu S et al. testified that mutant TP53 could rewire the tumor glycolytic metabolism and enhance metastasis in TME (Basu et al., 2018). Mutant TP53 has additional impacts on TME beyond changing tumor metabolic phenotypes. Myriad studies have indicated that mutant TP53 can remodel TME by several mechanisms. First, mutant TP53 could induce neo-angiogenesis by stimulating VEGF secretion (Kieser et al., 1994). Second, mutant TP53 could regulate chemokines that are involved in the homeostatic microenvironment (Yeudall et al., 2012). Last but not the least, mutant TP53 could reprogram the infiltrating immune cells and reshape the microenvironment (Cooks et al., 2018). TTN is located on chromosome 2q31, consisting of 364 exons, and it is the longest described coding gene (Chauveau et al., 2014). TTN ranks the third most abundant protein in both cardiac and skeletal muscle tissues, followed by actin and myosin (Chauveau et al., 2014). However, its mutation is not a rare entity in various cancers. Consistent with prior investigation by Cheng X et al., we also found that TTN missense mutation was a rather frequent type (Cheng et al., 2019). In addition, TTN and TP53 co-mutation is often accompanied during tumorigenesis and may serve as a prognostic biomarker either alone or in combination ^[61-63]^.

Current guidelines recommend adjuvant chemotherapy for patients at an advanced stage. However, how to select suitable patients who will benefit more from the chemotherapeutic regime is the prior concern. Our data demonstrated that patients with a high-risk score were more sensitive to camptothecin, gemcitabine, methotrexate, and vorinostat, suggesting that targeting glycolysis may alleviate the chemotherapeutic resistance. Despite immunotherapy bringing about a breakthrough for cancer patients, only a minority of patients could reap survival benefits actually. The TMB analysis in our study will accurately and effectively identify which patients will respond to immunotherapy in patients with EAC. Collectively, the proposed risk score system in our study has potency to help clinicians devise an individualized treatment strategy.

The strength of the present study is such that we performed a systematic analysis about glycolysis and TME in EAC for the first time based on the National Public Database, which provides robust data and statistical support. This study draws a close link between tumor glycolysis and the microenvironment and tentatively explains the mechanisms from the viewpoint of genetic alteration. Meanwhile, there are several limitations. First of all, TME is a complex mixture of parenchymal cells, the extracellular matrix, and numerous cytokines except for tumor and immune cells. These are not available from the public database and may greatly affect the analysis. Second, the results are not validated in *in vitro* and *in vivo* experiments. Last, the methods proposed in this study may not be applicable to all tumors as a result of heterogeneity. Notwithstanding its limitations, our study does provide an overview of glycolysis and TME in EAC, and this lays the foundation for further basic research in the area of metabolism and the microenvironment.

In summary, we found that glycolysis could change the microenvironment under the driver of genetic mutation and influence the immunotherapy in EAC. New efforts target that EAC should incorporate the idea that the glycolytic metabolism could reshape TME. Further studies are necessary to confirm our conclusion.

## Data Availability

The original contributions presented in the study are included in the article/Supplementary Material; further inquiries can be directed to the corresponding authors.

## Abbreviations

EAC, esophageal adenocarcinoma; TME, tumor microenvironment; TCGA, the cancer genome atlas; IICs, infiltrating immune cells; DEGs, differently expressed genes; FDR, false discovery rate; log2FC, log2 fold change; GSEA, gene set enrichment analysis; NOM, nominal; NES: normalized enrichment score; IGs, intersection genes; GO: gene ontology; KEGG, Kyoto encyclopedia of genes and genomes; BP, biological process; CC, cellular component; MF, molecular function; OS, overall survival; SNV, single nucleotide variant; TMB, tumor mutation burden; K-M, Kaplan–Meier; GDSC, genomics of drug sensitivity in cancer; TIDE, tumor immune dysfunction and exclusion; GEO, gene expression omnibus; VEGF, vascular endothelial growth factor; NPCs, nuclear pore complexes; HR, hazard ratio; SNP, single-nucleotide polymorphism; NPCs, nuclear pore complexes.

## Appendix A:

**TABLE A1:** R Glycolytic Effects in Other Cancers

Glycolysis factors	Tumor	Glycolytic effects
GRG	Bladder cancer	Influences cell proliferation and cycle
GRG	Breast cancer	Pro-tumor immunity
glycolysis	Cervical cancer	Indirect effect
SPAG4, ENO3, et al	Colon adenocarcinoma	Double effects on prognosis
GRG	Colorectal cancer	Double effects on prognosis
STC1, CLDN9, et al	Gastric cancer	Influence the microenvironment and prognosis
GGESS	Glioblastoma	Poor prognosis and poor chemotherapy
GRGPs	Hepatocellular carcinoma	Double effects on prognosis and treatments
GRG	HNSCC	Double effects on prognosis and treatments
HMMR, GPC1, et al	Lung cancer	Poor prognosis and metastasis
GRG	Ovarian cancer	Influence the microenvironment and therapy
glycolysis	Pancreatic cancer	Promote progression and reduce drug sensitivity
GRG	Prostate cancer	Cell migration and invasion inhibition
CD44, PLOD1, et al	Renal cell carcinoma	Tumor-promoting
GRG	Uveal melanoma	Influence the microenvironment, prognosis, and therapy

GRG: glycolysis-related gene; GGESS: glycolytic gene expression signature score; GRGPs: glycolysis-related gene pairs; HNSCC: head and neck squamous cell carcinoma.
